# Genetic relationships and low diversity among the tea-oil *Camellia* species in Sect*. Oleifera*, a bulk woody oil crop in China

**DOI:** 10.3389/fpls.2022.996731

**Published:** 2020-09-30

**Authors:** Huasha Qi, Xiuxiu Sun, Wuping Yan, Hang Ye, Jiali Chen, Jing Yu, Dai Jun, Chunmei Wang, Tengfei Xia, Xuan Chen, Dongliang Li, Daojun Zheng

**Affiliations:** ^1^Hainan, Academy of Agricultural Sciences, Sanya Institute, Sanya, China; ^2^Key Laboratory of Tropic Special Economic Plant Innovation and Utilization, National Germplasm Resource Chengmai Observation and Experiment Station, Institute of Tropical Horticulture Research, Hainan Academy of Agricultural Sciences, Haikou, China; ^3^School of Agricultural Sciences, Jiangxi Agricultural University, Nanchang, China; ^4^Guangxi Key Laboratory of Special Non-Wood Forest Cultivation and Utilization, Improved Variety and Cultivation Engineering Research Center of Oil-Tea Camellia in Guangxi, Guangxi Forestry Research Institute, Nanning, China; ^5^College of Horticulture, Hainan University, Haikou, China; ^6^Qionghai Tropical Crop Service Center, Qionghai, China

**Keywords:** tea-oil *Camellia*, *Camellia* Sect. *Oleifera*, germplasm resources, genetic relationships and diversity, classification

## Abstract

Tea-oil *Camellia* is one of the four woody oil crops in the world and has high ecological, economic and medicinal values. However, there are great differences in the classification and merging of tea-oil *Camellia* Sect. *Oleifera* species, which brings difficulties to the innovative utilization and production of tea-oil *Camellia* resources. Here, ISSR, SRAP and chloroplast sequence markers were analyzed in 18 populations of tea-oil *Camellia* Sect. *Oleifera* species to explore their phylogenetic relationships and genetic diversity. The results showed that their genetic diversity were low, with mean *H* and *π* values of 0.16 and 0.00140, respectively. There was high among-population genetic differentiation, with ISSR and SRAP markers showing an Fst of 0.38 and a high Nm of 1.77 and cpDNA markers showing an Fst of 0.65 and a low Nm of 0.27. The *C. gauchowensis*, *C. vietnamensis* and Hainan Island populations formed a single group, showing the closest relationships, and supported being the same species for them with the unifying name *C. drupifera* and classifying the resources on Hainan Island as *C. drupifera.* The tea-oil *Camellia* resources of Hainan Island should be classified as a special ecological type or variety of *C. drupifera*. However, cpDNA marker-based STRUCTURE analysis showed that the genetic components of the *C. osmantha* population formed an independent, homozygous cluster; hence, *C. osmantha* should be a new species in Sect*. Oleifera.* The *C. oleifera* var. *monosperma* and *C. oleifera* populations clustered into two distinct clades, and the *C. oleifera* var. *monosperma* populations formed an independent cluster, accounting for more than 99.00% of its genetic composition; however, the *C. oleifera* populations contained multiple different cluster components, indicating that *C. oleifera* var. *monosperma* significantly differs from *C. oleifera* and should be considered the independent species *C. meiocarpa.* Haplotype analysis revealed no rapid expansion in the tested populations, and the haplotypes of *C. oleifera*, *C. meiocarpa* and *C. osmantha* evolved from those of *C. drupifera*. Our results support the phylogenetic classification of *Camellia* subgenera, which is highly significant for breeding and production in tea-oil *Camellia*.

## Introduction

Within genus *Camellia* of family Theaceae, the more than 30 species of tea-oil *Camellia* are woody, oil-bearing tree species with a high content of seed oil that is widely processed into skin and health care products and edible oil (Chen et al., 2022). China is their place of origin and the main country where they are produced. Tea-oil *Camellia* is a major woody edible oil crop in China with a cultivated area of approximately 466 ha (Zhang et al., 2021). The tea-oil contained in the seed kernels of tea-oil *Camellia* fruit was listed as a healthy edible oil by the United Nations Food and Agricultural organization (FAO) in 2004. Tea oil is rich in squalene, vitamin E, *Camellia* glycosides and unsaturated fatty acids (Ma et al., 2011; Xiao et al., 2017). The content of unsaturated fatty acids in the edible oil is higher, reaching approximately 90%, and the content of oleic acid can reach approximately 87%. Tea oil is referred to as “Oriental olive oil” (Ye et al., 2020; Lin et al., 2022).

*Sect*. *Oleifera* belongs to genus *Camellia* of family Theaceae, and *C. oleifera*, *C. vietnamensis*, *C. gauchowensis*, and *C. oleifera* var. *monosperma* in this group are the main cultivated tea-oil species in China. At present, the main classification systems for *Camellia*, the Zhang (1998) system and the Min (1999) system, are based on morphology. Due to the morphological similarity among *Camellia* species and the rich diversity of the species in different regions, the two taxonomic systems provide different views regarding the taxonomic status of different *Camellia* species, including *Camellia* species in *Sect. Oleifera*. For example, there are five species and one variant of *Sect. Oleifera* under the Zhang Hongda system, including *C. gauchowensis*, *C. lanceoleosa*, *C. sasanqua*, *C. oleifera, C. vietnamensis* and *C. oleifera.* var. *monosperma*; however, there are 6 species under the Min Tianlu system in Flora of China (English version; Min and Bruce, 2007), in which *C. oleifera* var. *monosperma* is merged with *C. oleifera*, *C. gauchowensis* is combined with *C. vietnamensis* and these species are collectively referred to as *C. drupifera*. To clarify the taxonomic relationships among *Camellia* species, scholars have analyzed the phylogenetic relationships among different *Camellia* species using molecular taxonomy, such as taxonomies based on molecular markers (Shi et al., 1998; Vijayan et al., 2009; Li et al., 2020) and cpDNA (Yang et al., 2014, 2016), but there are no reports of the molecular evaluation and identification of the genetic relationships between *Sect. Oleifera* species. Disputes over classification have made the innovative utilization of tea-oil *Camellia* resources and production of tea-oil *Camellia* difficult.

Hainan Island is the southernmost edge of the distribution of tea-oil *Camellia* resources in China, characterized by a unique tropical-island climate appropriate for tea-oil *Camellia* and the isolation barrier formed by the Qiongzhou Strait (Ye et al., 2020). The tea-oil *Camellia* resources on Hainan Island are rich in significant morphological specialization (Zheng et al., 2016; Zhang et al., 2017). Our research showed that there are excellent tea-oil *Camellia* resources on Hainan Island with large fruit, thin skin and a high oil yield. The quality of tea oil from Hainan Island is also different from that in the main production areas in mainland China. These resources provide important genetic material for the breeding and improvement of tea-oil *Camellia* in China. Their excellent qualities have attracted considerable attention from researchers (Yao et al., 2019; Zhang et al., 2019; Xu et al., 2020; Ye et al., 2020, 2021). However, from the perspective of academia and production, the classification of tea-oil *Camellia* resources on Hainan Island is not clear.

Intersimple sequence repeat (ISSR) and sequence-related amplified polymorphism (SRAP) markers are two types of dominant markers that have been widely used in the identification and evaluation of plant populations (Hiremath et al., 2021; Song et al., 2021). Because of the maternal inheritance of plant cpDNA, it carries traces of the entire evolutionary history of plants (Ren et al., 2019), and cpDNA analysis is one of the main molecular methods used in studies of plant taxonomic identification and evolutionary origins (Almerekova et al., 2021; Hirota et al., 2021). In the present work, ISSR and SRAP markers and cpDNA sequence markers were used to study the genetic differentiation and relationships of *C. oleifera*, *C. vietnamensis*, *C. gauchowensis*, and *C. oleifera* var. *monosperma* along with *C. osmantha,* a new species of *Camellia,* and the tea-oil *Camellia* resources on Hainan Island. Our aims were: 1. to clarify the phylogenetic relationships among *C. oleifera, C. vietnamensis, C. gauchowensis* and *C. oleifera.* var. *monosperma*; 2. to determine whether *C. osmantha* can be considered a new independent species of Sect. *Oleifera*; 3. to identify and classify tea-oil *Camellia* resources on Hainan Island, including the species of these resources and whether they may represent an independent species or a specific taxonomic group within a species; and 4. to evaluate the genetic diversity and genetic differentiation among tea-oil *Camellia* resource populations from *Sect. Oleifera* species, including whether there is frequent gene flow among them.

## Materials and methods

### Plant materials collection

We collected samples from 18 populations, including four populations of *C. vietnamensis* and two populations of *C. oleifera.* var. *monosperma*, two populations of *C. oleifera*, two populations of *C. gauchowensis*, one population of *C. osmantha* and seven populations of tea-oil *Camellia* resources on Hainan Island, all of which are old seedling forest populations. The JXP and FY6 populations of *C. sinensis* from Jiangxi and Fujian, respectively, China, were used as an outgroup.

**Figure 1:**
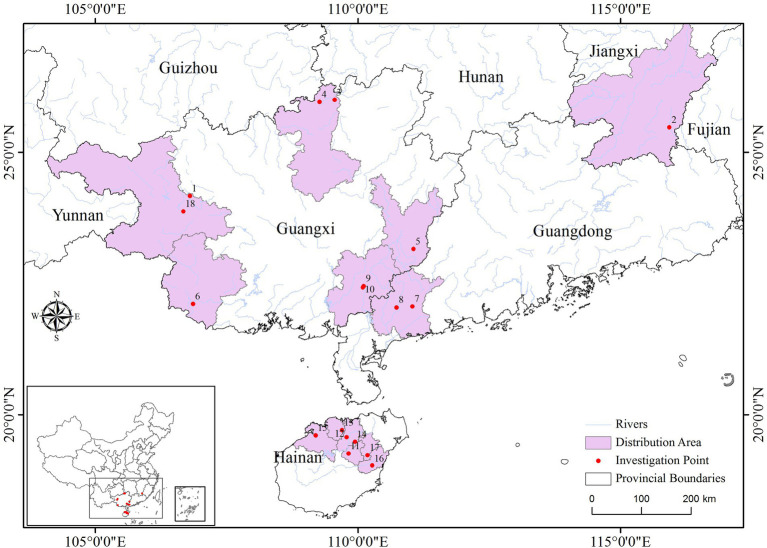
Sampling and distribution.

In the sampling of each population, the individual interval was greater than 15 meters. A total of 8 healthy young leaves were selected from each sample, washed with 75% alcohol, and immediately placed in silica gel for drying and sealing for subsequent molecular experiments. See Figure 1 and Supplementary Table 1 for basic information such as the number of individual plants collected and population information. During the chloroplast sequence analysis, five germplasm materials were randomly selected from each population.

### ISSR-PCR analysis

We performed ISSR-PCR to analyze 413 accessions from the 18 populations of *Camellia* spp. using 14 primers (Supplementary Table 2) and 2× San Taq PCR Mix (Sangon, Shanghai, China) following the manufacturer’s protocol. The PCR cycling conditions were as follows: initial denaturation at 94°C for 5 min; 40 cycles of denaturation at 94°C for 45 s, annealing at 53°C (or at primer-specific temperatures, see Supplementary Table 2) for 45 s and extension at 72°C for 1.5 min; with a final extension step at 72°C for 7 min.

### Sequence-related amplified polymorphism-PCR analysis

Sequence-related amplified polymorphism-PCR was performed to analyze 413 accessions from the 18 populations of *Camellia* spp. with 22 primer pairs (Supplementary Table 2). Following Qi et al. (2020), the PCR cycling conditions were as follows: initial denaturation at 94°C for 5 min; followed by 35 cycles of denaturation at 94°C for 60 s, annealing at 55°C (or at primer-specific temperatures, see Supplementary Table 2) for 60 s and extension at 72°C for 1.5 min; with a final extension step at 72°C for 7 min. Products were resolved on 1.5% agarose gels.

### Assessment of chloroplast sequence markers

Chloroplast genome sequencing was performed on all samples to screen single-base-variant ectopic sites and insertion/deletion sites. Five pairs of chloroplast gene fragment sequences were independently used. The amplified chloroplast gene fragment products were sequenced by Tianyihuiyuan Gene Technology Co., Ltd. Wuhan, China.

The amplification programs were as follows: pre-denaturation at 94°C for 5 min; 40 cycles of denaturation at 94°C for 1 min, annealing at 60°C for 1 min (annealing at primer temperatures, see Supplementary Table 3), and extension at 72°C for 1.5 min; and a final extension at 72°C for 10 min, followed by storage at 4°C. The volume of the reverse transcription reaction system was 30 μl, including 40 ng template DNA, 0.20 mmol/l dNTPs, 0.60 μMol/l primers, and 4.00U Taq DNA polymerase.

### Statistical analysis

#### Quantification of amplified bands

Clearly visible and repeatable bands were recorded as “1,” and the absence of a band at a particular position was recorded as “0.” Therefore, original matrices were established using the 0 and 1 scores. The amplification bands of each primer were counted, and the percentage of polymorphism (PPB) of each primer was calculated. The MXComp program in 02 software was used for the Mantel statistical test (1967) to analyze ISSR and SRAP data to compare the correlation between the two genetic similarity coefficient matrices.

#### Analysis of genetic diversity

Assuming valid Hardy–Weinberg equilibrium (HWE; Jubair et al., 2021; Graffelman and Weir, 2022; Yarar et al., 2022), we used POPGENE 1.32 software to analyze the original ISSR-PCR and SRAP-PCR data and determine genetic diversity indexes, including the percentage of polymorphic sites (P), observed number of alleles (Na), effective number of alleles (Ne), Shannon’s information index (I), and Nei’s gene diversity (H). DnaSP software was used to for the haploid analysis of 5 chloroplast sequences in different individuals and populations. The number of polymorphic sites (S), number of haplotypes (*h*), haplotype diversity (Hd), nucleotide diversity (*π*) and average number of nucleotide differences (K) were recorded.

To determine the degree of variation in genetic diversity among the test populations, the Na, Ne, H, I, and *p* values of the 18 populations were analyzed based on the coefficient of variation (CV), CV = (SD/mean) × 100%. To compare and evaluate the differences in genetic diversity among the populations, the Pearson correlation coefficients between ISSR markers and SRAP markers were analyzed using SPSS20 to verify the consistency of the results.

#### Relatedness and kinship analysis

To clarify the genetic relationships between populations and species from tea-oil *Camellia.* we performed the following steps: (1). Assuming that marker A was in Hardy–Weinberg equilibrium (HWE), POPGENE1.32 software was used to calculate the Nei’s genetic distance (GD) between the tested populations. (2). GenAlEx 6.5 software was used to analyze Fst and Nm based on cpDNA. (3). NTSYS-PC (2.02j) and MEGA6 were used to cluster the test populations by principal component analysis (PCA) and phylogenetic tree (Unweighted pair group method with arithmetic means, UPGMA tree; Neighbor-joining, NJ tree) analysis. (4) Based on the Bayesian selection allele frequency association model, STRUCTURE 2.3.2 was used to analyze the genetic composition of different individuals in the 18 tea-oil *Camellia* populations. After running this analysis, STRUCTURE 2.3.2 software was used to obtain the average L(K) value corresponding to each *K* value to determine the optimal *K* value (Evanno et al., 2005).

#### Phylogenetic relatedness and haplotype network analyses

DnaSP 5.0 was used to assess the number of haplotypes and the haplotype diversity of cpDNA in each group. A Neighbor-joining tree was constructed based on chloroplast haplotypes with MEGA6 software. The cpDNA haplotypes and their frequencies in each population are illustrated in pie charts. We used geographic information system (GIS) tools to map the sites of each population. NETWORK 5.001^1^ was used to construct a haplotype network diagram. The results were visualized by Cytoscape.

#### Analysis of historical population dynamics

To assess historical population dynamics, the mismatch distribution was estimated using DnaSP 5.0. For Tajima’s neutrality test, Tajima’s D values were tested for significant deviations from the null hypothesis of neutral evolution and constant population size.

## Results

### Primer amplification results among populations

A total of 198 bands were obtained from 14 ISSR primers, and the number of bands generated with each primer ranged from 11 to 17 (average 14.14), with an average percentage of polymorphism of 97.96% (91.67–100%). For SRAP markers, 291 bands were obtained, with an average PPB of 95.08% (76.92–100%), and the number of bands generated with each primer ranged from 8 (ME12-EM3) to 18 (ME8-EM1; average 9.18).

A total of five chloroplast sequences were obtained for this study, and the information of each sequence and its primers is shown in Supplementary Table 3. The aligned length of the five cpDNA sequence fragments was 2,971 base pairs (bp), with a total of 63 polymorphic sites (S), and the number of haplotypes (h) was 90 (Supplementary Table 3). The sequence fragment lengths of ZDJ43, ZDJ78, ZDJ87, ZDJ124 and ZDJ193 were 519, 726, 517, 616, and 593 bp, and they included 4, 23, 13, 17 and 6 polymorphic sites, respectively. The total *h* of the five aligned cpDNA sequence fragments was 53, ranging from 3 to 19, with an average of 10.60 (Supplementary Table 3).

### Genetic diversity of the 18 tested populations

The results of the Mantel test showed that there was a positive correlation between the genetic similarity coefficient matrices of ISSR and SRAP (*r* = 0.70). A correlation analysis (Pearson test) of the genetic diversity indexes of each population calculated based on the two marker types was carried out, and it was found that there was a significant positive correlation between the genetic diversity index data of the two markers, with Pearson correlation coefficients of 0.81 (*p* < 0.01) for Na and I with P, and 0.79 (*p* < 0.01) for both Ne and H. This showed that there was high consistency in the analyses of the 18 tested populations based on ISSR and SRAP markers. In the analysis and the discussion presented later in this paper, the comprehensive ISSR and SRAP data are used for analysis.

The results of ISSR and SRAP analyses showed that (Table 1), under Hardy–Weinberg equilibrium, the total genetic diversity of the tested populations was relatively high, with Na, Ne, H, I and *p* values of 1.97, 1.39, 0.25, and 0.39 and 96.52%, respectively, but the average level was lower, with Na, Ne, H, I and p values of 1.46, 1.28, 0.16, 0.24, and 46.47%, respectively. The ranges of H, I and PPB among the 18 populations were 0.13–0.22, 0.20–0.32, and 36.20–60.12%, respectively. The highest genetic variation was observed in the CKX population, with H, I and p values of 0.22, 0.32 and 60.12%, respectively, followed by the HCX population, whereas 7 populations on Hainan Island (JX, ZX, LS, XYL, MNL, MP, and FH) showed the lowest genetic variation, with H, I and p values lower than 0.14, 0.22 and 43.15%, respectively. The genetic variation levels among the tested populations were quite different, and the CV values of H, I and P were 16.32, 15.66 and 15.17%, respectively.

**Table 1:** Genetic diversity of the 18 tested populations of tea-oil *Camellia* resources in Sect. *Oleifera* based on ISSR, SRAP and chloroplast sequence markers.

Population	ISSR and SRAP					*Chloroplast* DNA						
	Observed number of alleles (Na)	Effective number of alleles (Ne)	Nei’s gene diversity (H)	Shannon’s information index (I)	Percentage of polymorphic sites (*P*, %)	Number of polymorphic sites (*S*)	Number of haplotypes(h)	Haplotype diversity (Hd)	Nucleotide diversity (*π*)	Theta-W	Average number of nucleotide differences (*K*)	Haplotype (frequencies, %)
HZP	1.57	1.34	0.20	0.30	56.85	22	5	1.000	0.00424	0.00356	12.600	H19(20), H20 (20),H21(20), H22(20), H23(20)
YML	1.47	1.29	0.17	0.25	46.83	22	4	0.900	0.00330	0.00356	9.800	H43(40), H50(20), H51(20), H52(20)
HCX	1.59	1.35	0.21	0.31	58.69	6	4	0.900	0.00101	0.00096	3.000	H6(20), H7(20), H12(20), H13(40)
CKX	1.60	1.37	0.22	0.32	60.12	5	4	0.900	0.00074	0.00081	2.200	H6(20), H7(20), H8(20), H9(40)
TPY	1.49	1.28	0.16	0.25	48.67	5	4	0.900	0.00074	0.00081	2.200	H42(20), H43(40), H44(20), H45(20)
BQY	1.52	1.31	0.18	0.27	51.74	8	5	1.000	0.00141	0.00129	4.200	H1(20), H2(20), H3(20), H4(20), H5(20)
WSG	1.49	1.29	0.17	0.26	48.67	6	5	1.000	0.00094	0.00097	2.800	H30(20), H41(20), H46(20), H47(20), H48(20)
TJG	1.45	1.27	0.16	0.23	45.40	7	5	1.000	0.00108	0.00113	3.200	H3(20), H39(20), H30(20), H40(20), H41(20)
LJTL	1.50	1.29	0.17	0.26	50.31	8	5	1.000	0.00148	0.00129	4.400	H2(20), H27(20), H28(20), H29(20), H30(20)
HTDL	1.45	1.25	0.15	0.22	44.99	35	5	1.000	0.00502	0.00566	14.900	H14(20), H15(20), H16(20), H17(20), H18(20)
JX	1.42	1.24	0.14	0.22	42.33	7	4	0.900	0.00135	0.00113	4.000	H10(40), H24(20), H25(20), H26(20)
ZX	1.43	1.26	0.15	0.22	43.15	2	3	0.800	0.00040	0.00032	1.200	H10(40), H31(20), H53(40)
LS	1.36	1.23	0.13	0.20	36.20	3	3	0.800	0.00047	0.00048	1.400	H10(40), H31(40), H32(20)
XYL	1.41	1.24	0.14	0.21	41.10	6	4	0.900	0.00114	0.00097	3.400	H10(20), H26(20), H35(20), H49(40)
MNL	1.39	1.24	0.14	0.21	39.26	1	2	0.400	0.00013	0.00016	0.400	H10(80), H31(20)
MP	1.38	1.23	0.13	0.20	37.83	5	4	0.900	0.00081	0.00081	2.400	H11(20), H33(40), H34(20), H35(20)
FH	1.39	1.24	0.14	0.20	39.06	3	2	0.400	0.00040	0.00048	1.200	H10(80), H11(20)
SNH	1.45	1.27	0.16	0.24	45.19	3	3	0.800	0.00047	0.00048	1.400	H36(40), H37(40), H38(20)
CV (%)	4.88	3.32	16.32	15.66	15.17	102.86	25.31	21.13	98.09	103.10	97.94	
Average	1.46	1.28	0.16	0.24	46.47							
Among Species	1.97	1.39	0.25	0.39	96.52							
*C. oleifera*	1.66	1.36	0.21	0.32	66.26	27	9	0.978	0.00413	0.00321	12.267	
*C. oleifera* var. *monosperma*	1.69	1.39	0.23	0.35	69.53	7	6	0.911	0.00094	0.00083	2.778	
*C. drupifera*	1.85	1.34	0.22	0.34	85.07	49	36	0.940	0.00220	0.00348	6.535	
*C. drupifera* on Hainan Island	1.72	1.29	0.18	0.29	72.39	9	12	0.803	0.00078	0.00074	2.319	
*C. drupifera* out Hainan Island	1.77	1.34	0.21	0.33	77.10	46	24	0.984	0.00309	0.00391	9.163	

The results of analyzing the five chloroplast sequences showed (Table 1) that the total genetic diversity level of the tested populations was low, the π and theta-W values were 0.00388 and 0.00418, respectively; and the average π and theta-W values were only 0.00139 and 0.00138, respectively. The HTDL population showed a higher S value of 35, with a π of 0.00502, indicating high genetic diversity. The next highest genetic diversity was observed for the HZP and YML populations, in which the S values were 22 and 22 and the π values were 0.00424 and 0.00330, respectively. However, the genetic diversity of the tested HCX, CKX, TPY, ZX, LS, MNL, MP and FH populations showed lower levels.

### Genetic differentiation among the 18 tested populations of tea-oil *Camellia* resources in Sect. *Oleifera*

The results of genetic differentiation analysis among the 18 tested populations (Table 2) showed that the genetic differentiation assessed based on ISSR and SRAP markers was lower among populations than within populations, and only 38% of the genetic variation occurred in the populations. At the cpDNA level, the genetic differentiation among populations was greater than that within populations, with 65% of differentiation being found among populations and 35% within populations, indicating that at the cpDNA level, most of the population genetic variation occurred between populations. Based on ISSR and SRAP analysis, Nm among populations was 1.77, indicating strong gene flow. However, in the chloroplast sequence analysis, Nm among populations was only 0.27, indicating that there was very low gene flow among populations at the cpDNA level.

**Table 2:** Genetic differentiation coefficients among the tested populations.

Population	nrDNA		cpDNA	
	Fst	Nm	Fst	Nm
All populations	0.38	1.77	0.65	0.27
*C. oleifera*	0.24	2.87	0.15	2.92
*C. oleifera* var. *monosperma*	0.14	4.59	0.11	4.06
*C. drupifera*	0.33	1.14	0.48	0.55
*C. drupifera* on Hainan Island	0.28	1.52	0.15	2.76
*C. drupifera* out Hainan Island	0.25	1.70	0.46	0.59
Between *C. oleifera* and *C. oleifera* var. *monosperma*	0.21	4.30	0.67	0.25
Between *C. oleifera* and *C. drupifera*	0.25	3.86	0.43	0.66
Between *C. oleifera* and *C. drupifera* on Hainan Island	0.34	2.74	0.63	0.30
Between *C. oleifera* and *C. drupifera* off Hainan Island	0.25	3.88	0.31	1.13
Between *C. oleifera* var. *monosperma* and *C. drupifera*	0.30	3.31	0.90	0.06
Between *C. oleifera* var. *monosperma* and *C. drupifera* on Hainan Island	0.39	2.32	0.90	0.06
Between *C. oleifera* var. *monosperma* and *C. drupifera* out Hainan Island	0.28	3.50	0.67	0.24
Between *C. osmantha* and *C. drupifera*	0.31	2.49	0.44	0.63

### Genetic relationship and structure analyses among the 18 tested populations of tea-oil *Camellia* resources in Sect. *Oleifera*

The Mantel test results showed that there was a significant positive correlation between the nuclear ribosomal DNA (ISSR and SRAP) and cpDNA genetic distance matrices (*r* = 0.72), with high consistency. In the ISSR and SRAP analyses (Supplementary Table 4), the Nei’s genetic distance (GD) between the tested populations varied from 0.026 to 0.199, with an average of 0.118. The HCX population of *C. oleifera* var. *monosperma* showed the most distant genetic relationship from the tea-oil *Camellia* resources on Hainan Island (MP and FH), with GDs of 0.198 and 0.199, respectively. In the cpDNA analysis, the GD between the tested populations varied from 0 to 0.009, with an average GD of 0.0028, while the GD between the populations on Hainan Island was only 0 or 0.001, and the genetic distance between the *C. oleifera.* var. *monosperma* populations and other populations was relatively large (generally 0.007).

**Figure 2:**
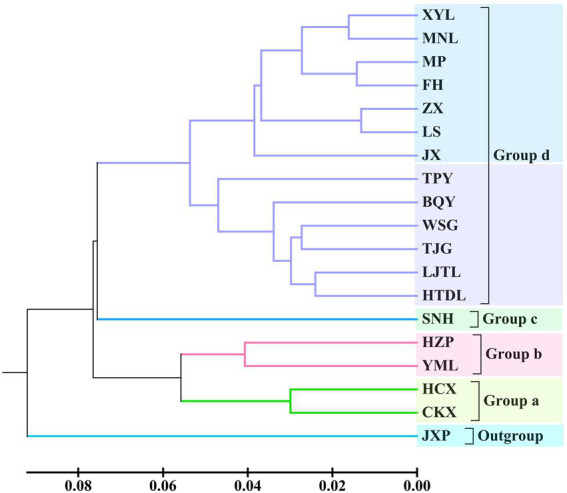
UPGMA dendrogram of the 18 tested populations based on ISSR and SRAP markers.

Based on the ISSR and SRAP data, a phylogenetic tree among the 18 tested populations was constructed *via* the unweighted pair group method with arithmetic means (UPGMA) method, and the 18 tested populations were grouped into four categories (Figure 2). Among these populations, two populations of *C. oleifera.* var. *monosperma* (GD = 0.060) and two populations of *C. oleifera* (GD = 0.081) were clustered into group a and group b, respectively. *C. osmantha* constituted group c, and its average GD from the other groups was 0.155, ranging from 0.129 to 0.182. Group d was composed of 13 populations of *C. gauchowensis* and *C. vietnamensis* and from Hainan Island. Seven populations on Hainan Island were first clustered into subgroup 4–1, and the genetic distance between the populations was relatively small, with an average GD of 0.065. Subcategory 4–2 was composed of a mixed cluster of the populations of *C. gauchowensis* and *C. vietnamensis*, with an average GD value of 0.107.

**Figure 3:**
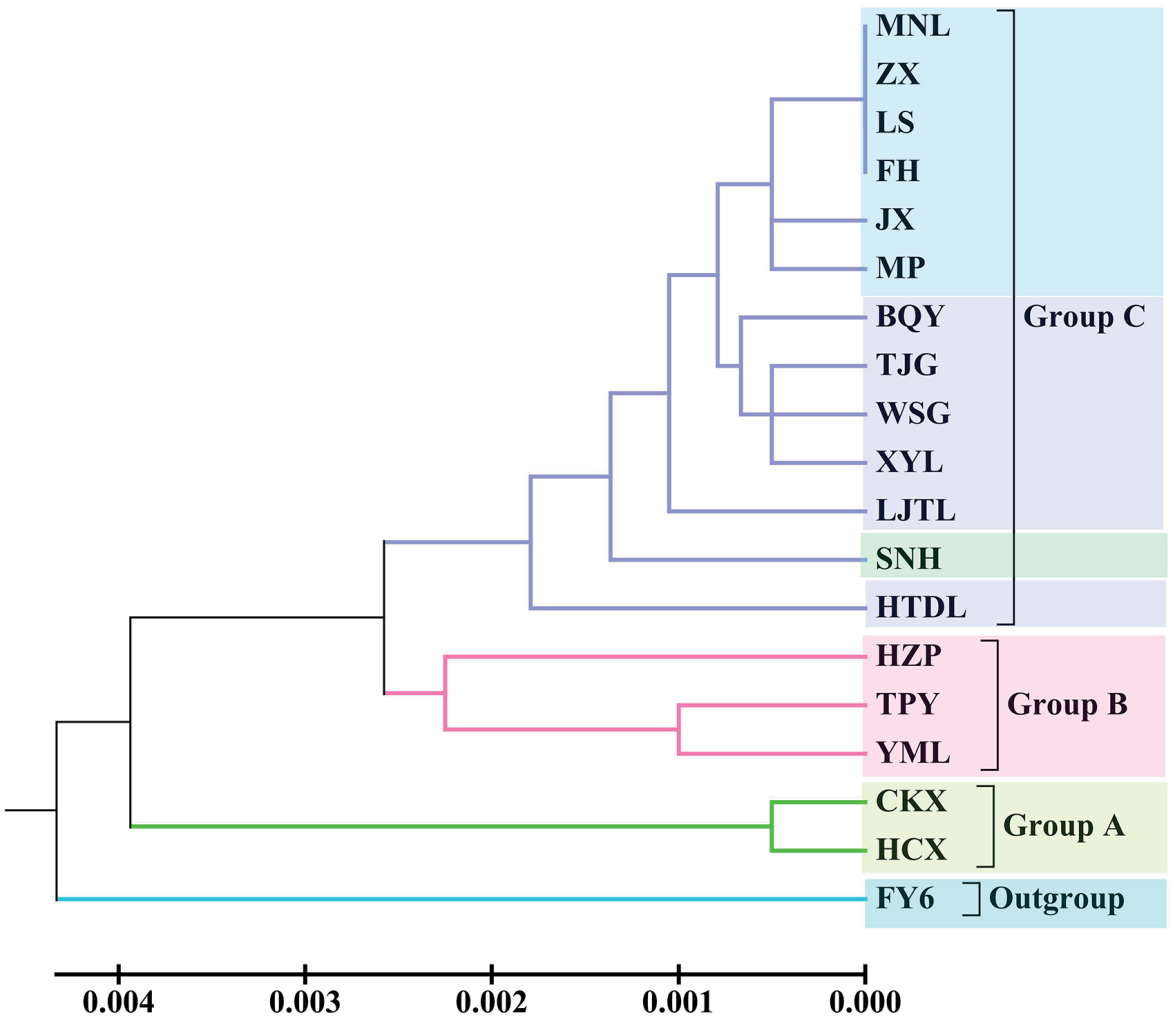
UPGMA dendrogram of the 18 tested populations based on cpDNA.

According to UPGMA tree clustering based on cpDNA, the 18 tested populations were clustered into three groups (Figure 3; Supplementary Table 4). Two populations of *C. oleifera.* var. *monosperma* constituted Group A. *C. oleifera* populations HZP, YML and TPY were included in Group B (GD = 0.001–0.003, average GD = 0.0017). Lastly, Group C consisted of *C. osmantha*, *C. gauchowensis*, *C. vietnamensis*, and populations on Hainan Island (GD = 0.0009), among which seven populations on Hainan Island were first clustered into a small subgroup.

**Figure 4:**
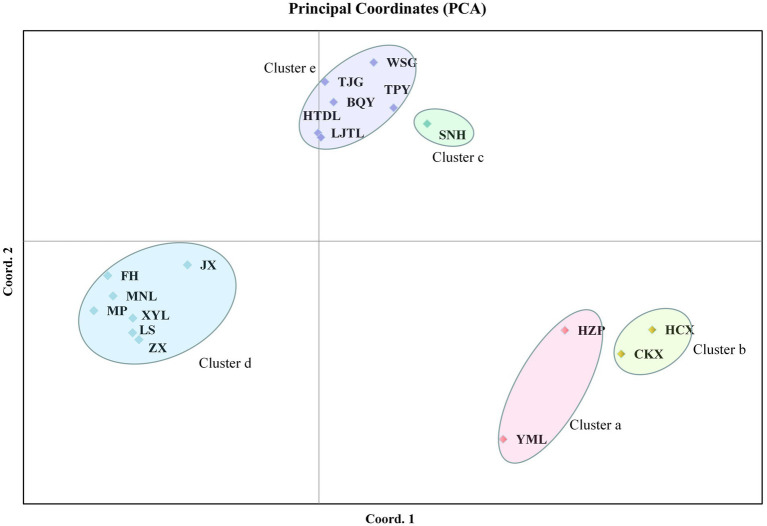
Principal component analysis (PCA) of the 18 tested populations based on ISSR and SRAP markers.

PCA of the ISSR and SRAP data showed that the tested populations could be divided into five clusters. The cumulative contribution of the first three principal components was 69.01% (Dim-1 = 35.06, Dim-2 = 17.62), indicating that the clustering results were reliable. Among these populations, *C. oleifera* (HZP and YML) and *C. oleifera* var. *monosperma* (HCX and CKX) were aggregated into Cluster a and Cluster b, respectively (Figure 4); Cluster c consisted solely of *C. osmantha* (SNH); Cluster d included all of the Hainan Island populations (XYL, MP, MNL, FH, ZX, LS, and JX); and Cluster e consisted of the *C. vietnamensis* and *C. gauchowensis* populations. In the two-dimensional plot, Cluster d was found to be located in the middle of all the classification groups, which is the closest to Cluster e. The clustering results were basically consistent with the UPGMA trees constructed for all 18 tested populations.

**Figure 5:**
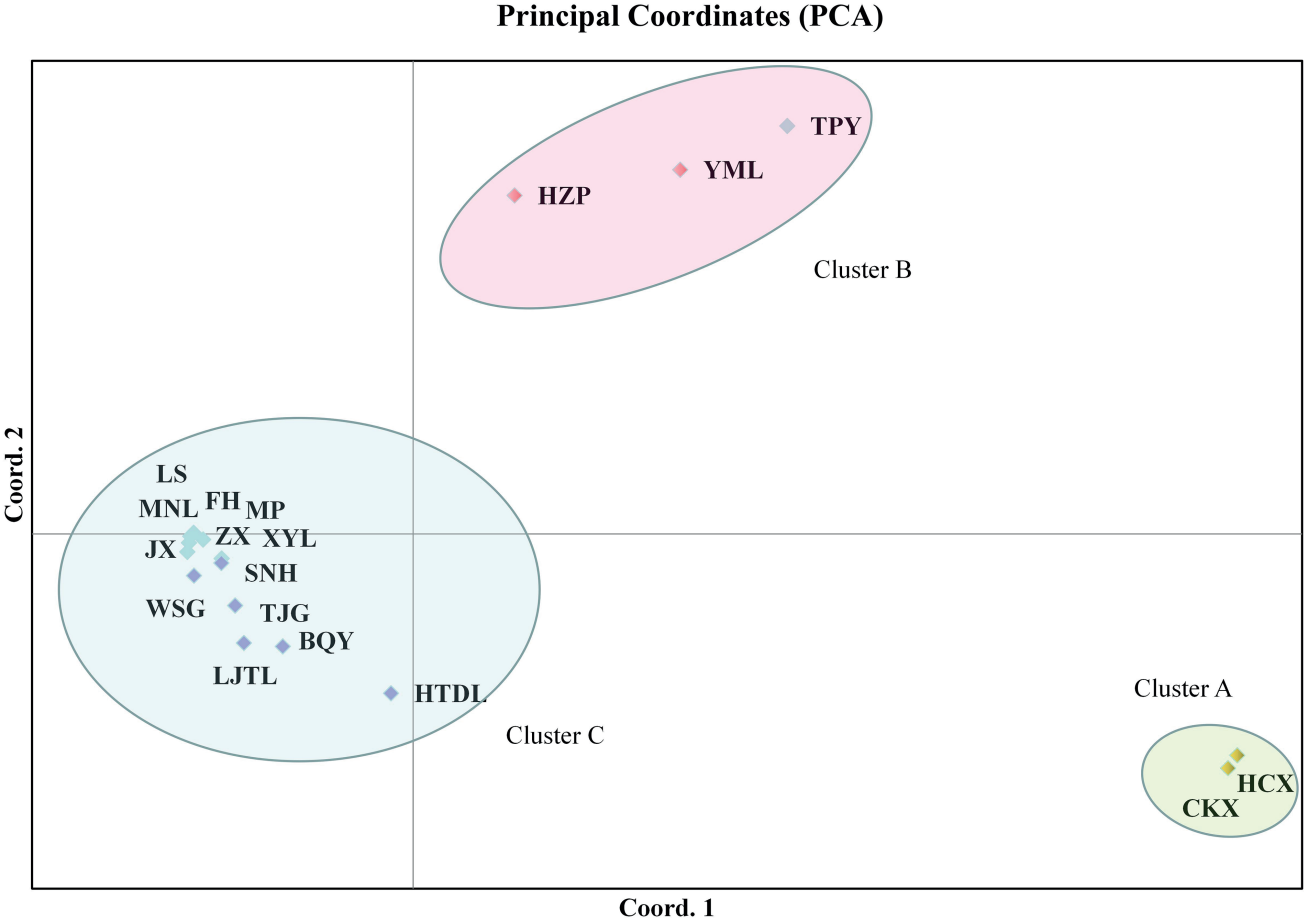
Principal component analysis (PCA) of the 18 tested populations based on cpDNA.

The results of PCA based on cpDNA showed that the tested populations could be divided into four clusters. The cumulative contribution of the first three principal components was 82.50% (Dim-1 = 53.14, Dim-2 = 17.47), indicating that the clustering results were reliable. The two populations of *C. oleifera.* Var. *monosperma* (HCX and CKX) were grouped into Cluster A (Figure 5); Cluster B consisted of TPY, HZP and YML; and Cluster C included populations in Hainan, *C. osmantha* (SNH), *C. vietnamensis* and *C. gauchowensis*.

According to PCA clustering, the TPY population tended to belong to the *C. vietnamensis* cluster based on the ISSR and SRAP markers but to the *C. oleifera* cluster (cluster C) according to the cpDNA data.

**Figure 6:**
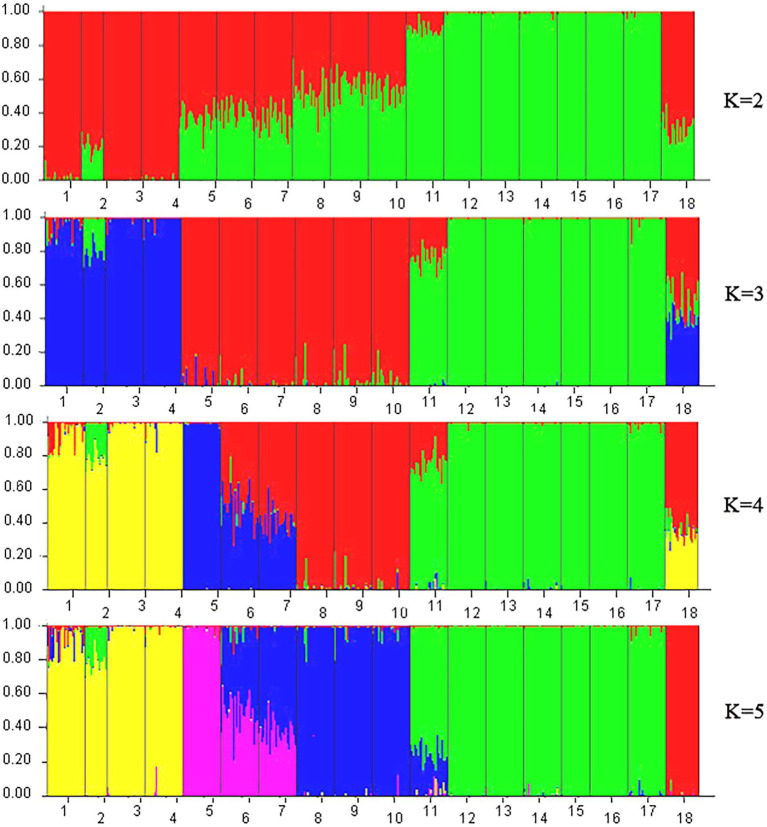
STRUCTURE analysis based on Bayesian clustering according to ISSR and SRAP data. Nos. 1–18 represent populations HZP, YML, HCX, CKX, TPY, BQY, WSG, TJG, LJTL, HTDL, JX, ZX, LS, XYL, MNL, MP, FH, and SNH, respectively.

**Figure 7:**
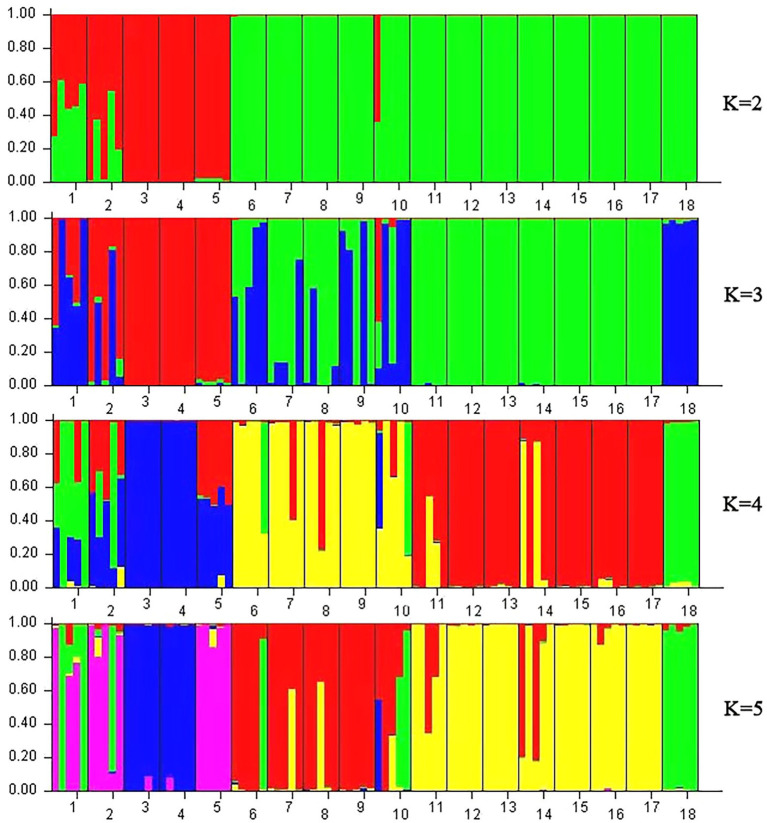
STRUCTURE analysis based on Bayesian clustering according to cpDNA data. Note: Nos. 1–18 represent populations HZP, YML, HCX, CKX, TPY, BQY, WSG, TJG, LJTL, HTDL, JX, ZX, LS, XYL, MNL, MP, FH, and SNH, respectively.

A model-based Bayesian cluster analysis was performed in STRUCTURE to visualize the genetic structure of the 18 tested populations. The results showed that the delta K value was largest when the K value was 2 (Supplementary Figures 1, 2), indicating that *K* = 2 was the best value and that all the tested materials could be divided into two clusters: Cluster 1 and Cluster 2 (Figures 6, 7). Clusters 1 (red) and 2 (green) represent the different genetic material contributed by each population.

For the ISSR and SRAP data, when *K* = 2, 6 populations among the seven populations from Hainan Island accounted for more than 99.1% of the genetic material from Cluster 2, and only 89.5% of the genetic material in the JX population came from Cluster 2 (Figure 6). A total of 98.4 and 80.0% of the genetic material of HZP and YML individuals, respectively, in the *C. oleifera* populations was classified into Cluster 1; more than 99.2% of the genetic material of the HCX and CKX individuals in the *C. oleifera* var. *monosperma* populations came from Cluster 1. Approximately 50.0% of the genetic material of the *C. vietnamensis* and *C. gauchowensis* individuals came from Cluster 1 and Cluster 2, respectively. A total of 69.6% of the genetic material of the *C. osmantha* individuals came from Cluster 1, and 30.4% came from Cluster 2. These results indicate that when *K* = 2, the genetic material of individuals of the Hainan Island populations and *C. oleifera.* var. *monosperma* is simple, while the genetic information of individuals of the *C. osmantha*, *C. vietnamensis* and *C. gauchowensis* populations is complex. When *K* = 3, the genetic components of *C. oleifera* and *C. oleifera.* var. *monosperma* came mainly from Cluster 3, and a small amount of the genetic components of *C. oleifera* individuals came from Cluster 1 and Cluster 2. The main genetic components of Cluster 1 and Cluster 2 came from the *C. gauchowensis*, *C. vietnamensis*, and Hainan populations. When *K* = 4 and *K* = 5, the main genetic components of the BQY and WSG populations came from *C. oleifera* (37.2–58.6%), showing significant complexity; the HCX and CKX individuals of *C. oleifera.* var. *monosperma* were highly homozygous, and the majority of the genetic material of *C. oleifera* HZP and YML individuals came from the same cluster as *C. oleifera.* var. *monosperma* but was mixed with other cluster components. When *K* = 4 and *K* = 5, the seven tested populations on Hainan Island showed a high degree of homozygosity, and 6 populations presented more than 97.5% homozygosity; JX represented the sole exception, showing 73.0% (*K* = 4) and 74.4% (*K* = 5) homozygosity. *C. osmantha* presented significant complexity when *K* = 2–4, as nearly 50% of the genetic components came from two different clusters, whereas when *K* = 5, it was 99.2% homozygous.

Based on the cpDNA data (Figure 7), when *K* = 2, while the genetic material of *C. oleifera* individuals (HZP and YML populations) were diverse and accounted for between 23.1 and 76.9% of the material in Cluster 1 and Cluster 2, respectively, the genetic components of the remaining 16 populations came from a single source. The individuals of HCX, CKX and TPY accounted for more than 99.7% of the genetic material from Cluster 1, and those of BQY, WSG, TJG, LJTL, HTDL, JX, ZX, LS, XYL, MNL, MP, FH and SNH accounted for more than 86.8% of the genetic material from Cluster 2. When *K* = 3, the genetic components of the HCX, CKX and TPY populations, the Hainan populations, and the *C. osmantha* population came from three different clusters, showing a high degree of homozygosity (more than 97.2% of the genetic material). The genetic components of the *C. oleifera* population came mainly from the populations of *C. oleifera.* var. *monosperma* and *C. osmantha*, while the genetic components of the BQY, WSG, TJG, LJTL, and HTDL populations came mainly from the Hainan and *C. osmantha* populations, all of which showed significant complexity. When *K* = 4 and *K* = 5, the population genetic material of *C. oleifera*, *C. gauchowensis* and *C. vietnamensis* was observed to be complex and contained genetic components from the Hainan Island, *C. oleifera.* var. *monosperma* and *C. osmantha* populations. Among the Hainan Island populations, genetic exchange was observed only between *C. gauchowensis* and *C. vietnamensis*. Similarly, the populations of *C. oleifera.* var. *monosperma* and *C. osmantha* maintained relatively high homozygosity of their genetic components. When *K* = 4, the genetic material of HCX, CKX and SNH accounted for 99.6, 99.6 and 96.2%, respectively, whereas when *K* = 5, the genetic material of HCX, CKX and SNH accounted for 97.5, 97.5 and 96.4%, respectively.

### Haplotype phylogenetic relationships among the 18 tested populations

**Figure 8:**
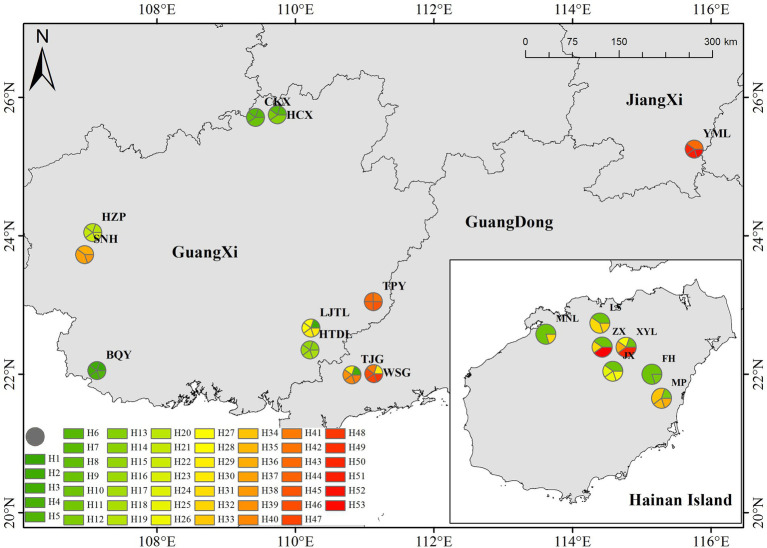
Distribution of cpDNA haplotypes in 53 ribotypes of the test populations.

**Figure 9:**
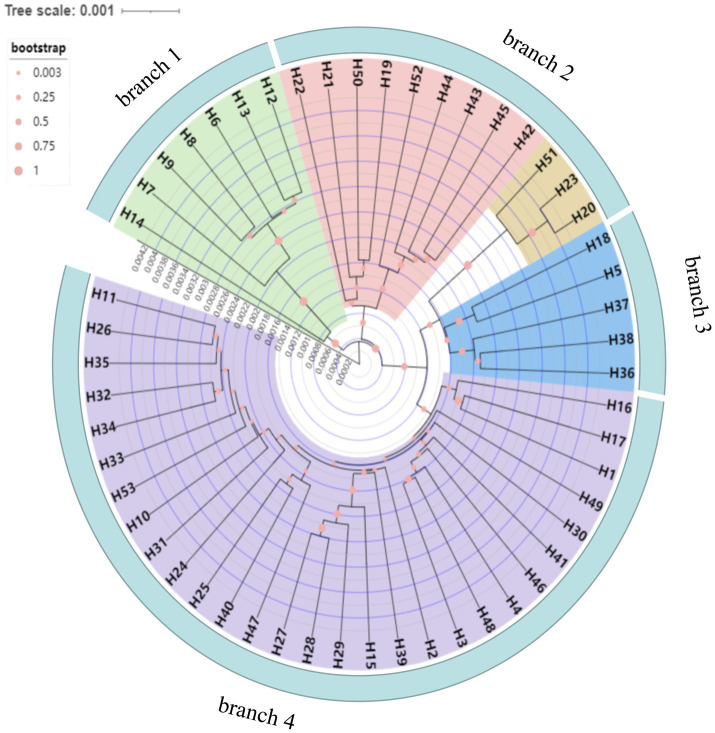
Neighbor-Joining (NJ) dendrogram of the 53 cpDNA haplotypes of the test populations.

**Figure 10:**
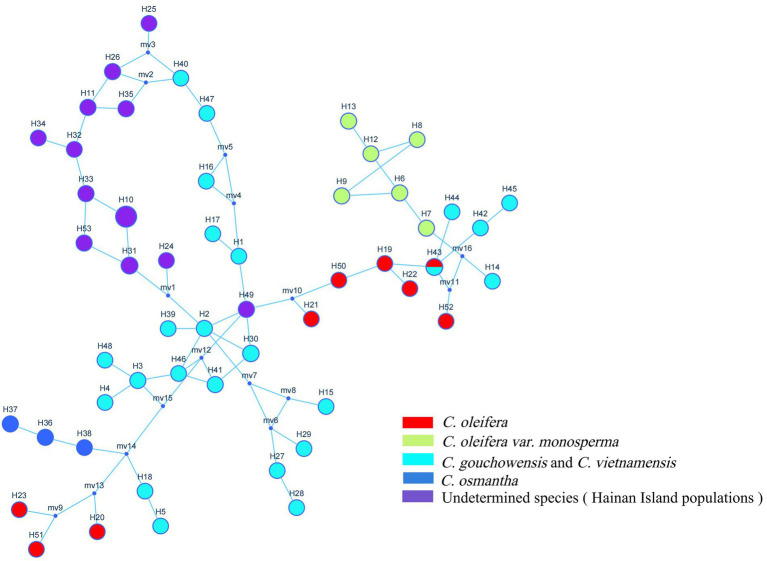
TCS network of the 53 cpDNA haplotypes of the test populations.

Fifty-three haplotypes were detected among the five combined cpDNA sequences of the test population, and the geographic distribution, phylogenetic tree and topological structure of the 53 haplotypes were constructed (Figures 8–10). In the phylogenetic tree (NJ tree), the 53 haplotypes could be divided into four branches. Haplotypes H6 to H9 and H12 to H14 were clustered into branch 1, in which all haplotypes except for H14 from the HTDL population came from the *C. oleifera* var. *monosperma* HCX and CKX populations, located in northern Guangxi (Figure 8). The haplotypes that made up branch 2 were all derived from a subset of the haplotypes of the HZP and YML populations of *C. oleifera* and all haplotypes of the TPY population. Branch 3 consisted of two subbranches, in which all haplotypes from SNH, H18 of HTDL and H5 of BOY formed subbranch A, and subbranch B was composed of H20, H23 of HZP and H51 of YML. Branch 4 included all haplotypes of the Hainan, *C. gauchowensis* and *C. vietnamensis* populations except for H5 of BOY and H18 and H14 of HTDL. All of these haplotypes except for H49 in the Hainan population are clustered into a small subbranch located at the bottom of the phylogenetic tree. Finally, the relationships between the ancestral and derived cpDNA haplotypes were also analyzed in a TCS network diagram (shown in Figure 10).

According to the positions of the haplotypes in the network (Figure 10), H2 located in a central position within the network, was inferred to be the ancestral haplotype, and H12 and H26 form a small radiation. Most of the remaining haplotypes showed only one or two mutations relative to the other haplotypes. According to the geographical distribution of the haplotypes (Figure 8), haplotypes H30, H31 and H43 were shared in three different populations, and H2, H3, H6, H7, H11, H26, H3, H35 and H41 were shared in two different populations. H10 was the most widely shared haplotype, being distributed in six populations, FH, JX, LS, MNL, XYL, and ZX, so it was inferred to be the ancestral haplotype. The *C. vietnamensis* (H2) and Hainan (H10) populations could be the origin of the ancestral haplotype.

### Analysis of the historical dynamics of the test populations

**Figure 11:**
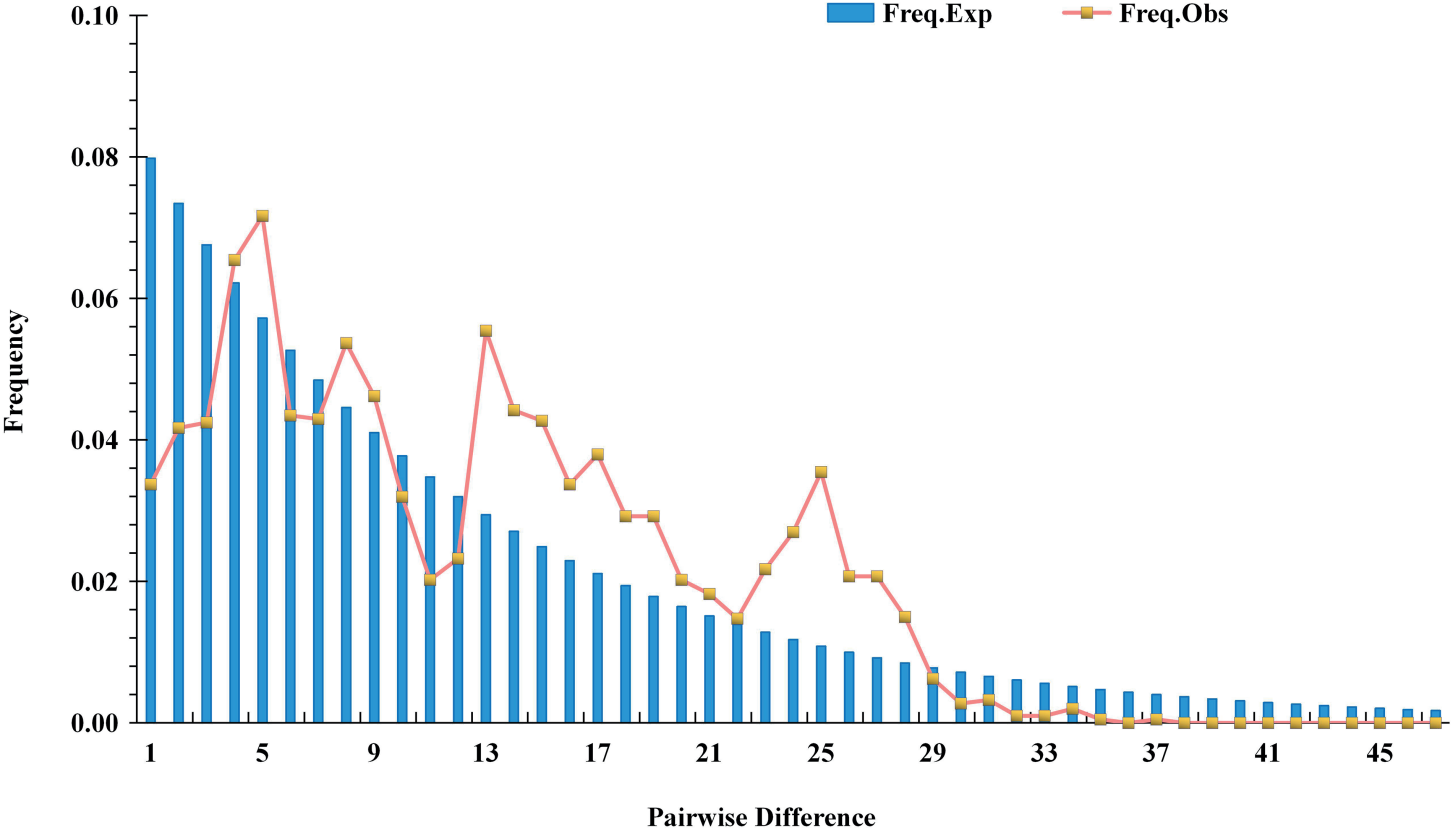
Mismatch distribution plots for the test populations.

Based on cpDNA sequence data, DnaSP software was used to analyze the mismatch distribution of the tested populations to determine whether the tested population experienced a rapid expansion event (Figure 11; Supplementary Table 5). The results showed no single peak in the distribution of chloroplast gene mismatches (Freq. Exp), indicating that the tested populations represented a model of recalcitrance to expansion, having experienced no obvious expansion. The results of Tajima’s neutrality test for the five chloroplast fragments showed a Tajima’s *D* value of −0.41555 (*p* > 0.10) and an Fu’s Fs value of −0.51948, and the Tajima’s *D* values were not significantly negative. These results indicated that all of the tested loci presented neutral evolution and that the tested populations had not experienced a rapid expansion event. This was completely consistent with the results of the mismatch distribution analysis.

## Discussion

### Relationships among the tea-oil *Camellia* species from Sect*. Oleifera*

#### The classification of *C. oleifera.* var. *monosperma*

According to the Zhang Hongda system in Flora of China (Chinese version; Zhang, 1998), *C. oleifera.* var. *monosperma* is a variant of *C. oleifera.* In Flora of China (English version; Min and Bruce, 2007), *C. oleifera.* var. *monosperma* was a synonym, rather than a variant of *C. oleifera*. In this study, the results of the UPGMA dendrogram analysis and PCA based on either ISSR and SRAP data or cpDNA genetic information divided the *C. oleifera* var. *monosperma* populations and *C. oleifera* populations into significantly different groups (Figures 2–5). The two populations of *C. oleifera* var. *monosperma* in the PCA graph are located on the far right according to the one-dimensional coordinates. The genetic differentiation between the *C. oleifera* var. *monosperma* and *C. oleifera* populations based on cpDNA was especially large, with an *F*st value of 0.67 and a gene flow value of 0.251 (Table 2). In the STRUCTURE analysis based on either ISSR and SRAP or cpDNA genetic information, when *K* = 2–5, the *C. oleifera.* var. *monosperma* populations were divided into an independent group accounting for more than 99.00% of the genetic composition, while the *C. oleifera* populations always contained genetic compositions from different clusters (Figures 6, 7). According to the cpDNA marker analysis in particular, the genetic components of the *C. oleifera* populations came from multiple clusters. For example, for *K* values from 2 to 4, the average percentages of individual genetic material in the *C. oleifera* populations were 64.80, 48.05 and 29.25%, respectively, and when *K* = 5, no genetic components of *C. oleifera.* var. *monosperma* were observed. Furthermore, five chloroplast sequence haplotypes were detected in the two *C. oleifera.* var. *monosperma* populations, which were of the same origin, clustered into the same branch and showed no crossing with the haplotypes of the other tested populations. According to our field survey results and the description in the Flora of China (Chinese version), *C. oleifera.* var. *monosperma* exhibits small leaves, small flowers, small fruit, mostly single seeds, a thin peel and a high oil content of its fruit, which are significantly different from the characteristics of *C. oleifera*. *C. oleifera.* var. *monosperma* is the second most commonly cultivated tea-oil *Camellia* species in China after *C. oleifera* and is mainly distributed in Fujian, Jiangxi, and Guangxi. Most producers and researchers refer to this species as *C. meiocarpa*.

Therefore, regardless of the above molecular analysis results, phenotypic differences, and industrial development and research needs, *C. oleifera* var. *monosperma* should be considered an independent species named *C. meiocarpa* (in the following section, both are called *C. meiocarpa*). Thus, it should not be simply classified within *C. oleifera* (Min and Bruce, 2007), which is consistent with the findings of Huang (2011), Liu (2010), and Xie (2013).

#### The relationship between *C. vietnamensis* and *C. gauchowensis*

In the Flora of China (Chinese version), *C. vietnamensis* and *C. gauchowensis* are located in relatively distant taxonomic positions, relative to classification status of *C. vietnamensis* and *C. oleifera* (Zhang, 1998). In this study, both the UPGMA clustering and PCA results showed that the *C. gauchowensis* and *C. vietnamensis* populations were included in the same group (Figures 2–5), with a low average GD of 0.072 according to ISSR and SRAP markers and a low average GD of 0.0018 according to cpDNA markers (Supplementary Tables 2, 3). The genetic material components of these two species were also relatively consistent in the STRUCTURE analysis. These results indicated that the populations of *C. gauchowensis* and *C. vietnamensis* presented the closest relationship.

One key morphological difference between *C. gauchowensis* and *C. vietnamensis* in morphological taxonomy is whether or not current-year branchlets are glabrous. Under the Zhang Hongda system in Flora of China (Chinese version), the current-year branchlets and leaves of *C. gauchowensis* are glabrous, while they are gray-brown pubescent for *C. vietnamensis.* Under the Min Tianlu system in Flora of China (English version), the current-year branchlets of both *C. gauchowensis* and *C. vietnamensis* (both of which are revised names of *C. drupifera*) are glabrous (Min and Bruce, 2007). However, our field survey data showed that most of the branchlets were hairy in all *C. gauchowensis* and *C. vietnamensis* populations, and only a few populations had individual plants with hairless branchlets. Therefore, based on the above analysis results, we believe that *C. gauchowensis* and *C. vietnamensis* should be merged into the same species and renamed *C. drupifera*, which is supported by the Flora of China (English version; Min and Bruce, 2007). However, this conclusion is inconsistent with the analysis results by Xie (2013) based on AFLP markers, suggesting that *C. gauchowensis* is more closely related to *C. oleifera* than *C. vietnamensis*.

#### Relationships between *C. osmantha* and the other tea-oil species from Sect. *Oleifera*

*C. osmantha* was once considered a variant of *C. oleifera* due to the similarity of its leaf and bud morphology to that of *C. oleifera*. According to a morphological and taxonomic evaluation conducted by Ye et al., *C. osmantha* is a new species belonging to Sect. *Paracamellia* of *Camellia*. Its main morphological features are short columns, slightly fragrant flowers, small fruit and many single seeds (Ma et al., 2012). Due to the large plant size, high fruit yield, thin fruit skin and high oil content of *C. osmantha*, it has been promoted as the main cultivated species in Guangxi, China. The results of UPGMA clustering and PCA based on ISSR and SRAP data in this study showed that the *C. osmantha* population was an independent group located between *C. oleifera* and *C. drupifera*, with a closer relationship to *C. drupifera* (GD = 0.1665 for *C. osmantha* with *C. oleifera* and GD = 0.1510 for *C. osmantha* with *C. drupifera*; Figures 2, 4; Supplementary Table 4).

According to the cpDNA data, *C. osmantha* was clustered with *C. drupifera* in the phylogenetic tree and two-dimensional PCA map (Figures 3, 5), but the genetic distance between *C. osmantha* and all *C. drupifera* populations (average GD = 0.0024) was significantly larger than that among the *C. drupifera* populations (average GD = 0.0012; Supplementary Table 4). Furthermore, in a STRUCTURE analysis performed with *K* values of 2 to 4, *C. osmantha* was a mixed component of *C. meiocarpa* and *C. drupifera*, whereas when *K* ≥ 5, *C. osmantha* formed an independent cluster (more than 99% of genetic components) according to ISSR and SRAP data (Figure 6). According to the cpDNA data, when *K* = 2, *C. osmantha* belonged to the same cluster as *C. drupifera* (more than 99% of genetic components); at *K* values of 3 to 5, *C. osmantha* formed an independent cluster (more than 99% of genetic components; Figure 7). Considering the results of the morphological and molecular data analysis as well as tea-oil production requirements, we suggest that *C. osmantha* can be classified as an independent taxonomic unit, it is closely related to *C. drupifera* and it belongs to Sect*. Oleifera* of the *Camellia* genus.

#### Molecular identification of tea-oil *Camellia* germplasm resources on Hainan Island

The phylogenetic tree and PCA results of this study showed that all of the Hainan populations were clustered with the *C. drupifera* populations and that they presented the closest relationship with *C. drupifera* among all the tested populations (mean GDs from *C. drupifera* populations, *C. oleifera* populations, *C. meiocarpa* populations, and *C. osmantha* populations of 0.1069, 0.1504, 0.1768, and 0.1640, respectively, according to ISSR and SRAP data and 0.0016, 0.0030, 0.007, and 0.0022 according to cpDNA data). Further analysis based on cpDNA, ISSR and SRAP data showed that the populations from Hainan Island (except for the XYL population in the cpDNA data analysis) clustered together first and were located at the very edge in both the phylogenetic tree and PCA results (Figures 2–5). A STRUCTURE analysis conducted with *K* = 2–5 showed that the populations from Hainan Island were all located in independent clusters with high genetic component percentages (approximately 98.0%, except for JX and XYL, which presented values of approximately 75.0%). Accordingly, we suggest that the tea-oil *Camellia* resources on Hainan Island belong to *C. drupifera*.

There was a closer genetic relationship among the populations from Hainan Island, with mean GDs of 0.065 based on nrDNA and 0–0.001 based on cpDNA, and they showed a relatively simple genetic background, with the lowest genetic diversity identified (average *H* of only 0.138, average π of 0.0047) and high gene flow (Nm = 1.52 for ISSR and SRAP, Nm = 2.76 for cpDNA). These populations exhibited significant genetic differentiation from and limited gene flow with the populations of *C. drupifera*, *C. oleifera*, *C. meiocarpa*, with cpDNA-based Nm values of 0.55, 0.298 and 0.057, respectively (Table 2). The above analysis showed that the tea-oil *Camellia* resources on Hainan Island constitute a special group that differs from all of the tested off-island populations. This difference may be caused by the homogenized habitat conditions on the island (Binks et al., 2019; Gai et al., 2021) as well as the limited hybridization and genetic introgression with off-island tea-oil *Camellia* resources due to the isolation of the island (Li et al., 2020). Therefore, we suggest that the Hainan tea-oil *Camellia* resources belong to a special ecological type or variant of *C. drupifera*. Based on some significant phenotypic differences and molecular information, Xu et al. (2020) identified a new species of tea-oil *Camellia* on Hainan Island, named *C. hainanica*.

### Genetic diversity of tea-oil *Camellia* germplasm resources from Sect. *Oleifera* of *Camellia*

Nybom (2004) counted 60 papers applying RAPD markers and four applying ISSR markers in plants and found that the average H value of the examined plants was 0.22. The H values of the 18 populations of the present study revealed by ISSR and SRAP markers ranged from 0.13 to 0.22, with an average of 0.16, indicating that the tested populations showed low genetic diversity (Table 1). The π values of the tested populations revealed by the five chloroplast sequences varied widely, ranging from 0.00013 to 0.00502, with an average value of 0.00140 (Table 1). This is similar to the levels of genetic diversity observed in other *Camellia* species, such as *Camellia huana* (π of 0.00042; Li et al., 2020), *Camellia flavida* (*π* of 0.00157; Wei et al., 2017), and *Camellia nitidissima* (π of 0.00082; Lu et al., 2020). However, the π values of most species are between 0.0020 and 0.0058 (Chen et al., 2012; Xing et al., 2017), relative to which the genetic diversity level of the population tested herein is low.

In this study, the genetic diversity index differed greatly among the 18 populations. The CV values of the *H*, *I*, *π* and Theta-*W* indexes between the populations were 16.32, 15.66, 98.09 and 103.10%, respectively (Table 1). The genetic diversity of the *C. oleifera* population was found to be relatively high regardless of whether the analysis was based on ISSR and SRAP or cpDNA data, with average *H* and *π* values of 0.185 and 0.00377, respectively, while the population on Hainan Island showed the lowest genetic diversity, with average *H* and *π* values of only 0.14 and 0.00067, respectively. *C. oleifera* shows the largest distribution and cultivation areas among tea-oil *Camellia* species, and there are frequent opportunities for gene exchange with multiple closely related species in the same area, resulting in the high genetic diversity of *C. oleifera*. In addition, the diverse habitat conditions in various regions have a positive impact on the high genetic diversity of this species (Feng et al., 2014). As mentioned above, the low genetic diversity of the populations on Hainan Island may be related to the lack of opportunities for gene exchange and introgression caused by island isolation as well as natural selection within the homogeneous habitat of the island (Li et al., 2020).

### Genetic differentiation and communication within Sect. *Oleifera* of *Camellia*

It is generally believed that an Fst > 0.25 indicates relatively high genetic differentiation among populations (Wright, 1978). In the present study, analyses based on nrDNA markers (ISSR and SRAP markers) and cpDNA sequence markers revealed Fst values of 0.38 and 0.65, respectively, among the tested populations, indicating high genetic differentiation among the tested populations (Table 2). According to analyses based on ISSR and SRAP markers, the majority of genetic variation exists within the populations (62%), which is consistent with the existence of abundant phenotypic variation within a population (Yang et al., 2018; Xia et al., 2022). Among the tested populations, Nm was 1.77 based on ISSR and SRAP analysis (Table 1), and this value greater than 1.00 indicated good gene exchange between the populations (Slatkin, 1987). This finding may be explained by the frequent gene exchange caused by the good hybrid affinity between the tested species during their introduction and cultivation and the homogenization of artificial selection during the cultivation process (Lu et al., 2015). cpDNA is maternally inherited and evolves slowly; the gene flow of cpDNA among the tested populations was low, with an Nm of only 0.25, and the majority of genetic variation occurred between the populations (up to 65%). The gene flow between the populations of *C. meiocarpa* and *C. drupifera* was only 0.06, with a differentiation coefficient up to 0.90, while the degree of genetic differentiation between *C. oleifera* and *C. drupifera* (Fst = 0.43; Nm = 0.66) was higher than that between the *C. oleifera* and *C. meiocarpa* (Fst = 0.67; Nm = 0.25) low. Further analysis showed that the genetic distance between the tested populations was positively correlated with geographic distance (r was 0.7590 for ISSR and SRAP markers and 0.5488 for cpDNA markers), revealing that geographic distance factors play a role in population genetic analysis and gene exchange.

### Preliminary study of the origin and evolution of tea-oil *Camellia* germplasm resources from Sect. *Oleifera*

cpDNA sequences provide important information for analyzing the origin and evolution of resources because of their maternal inheritance and good evolutionary conservation (Chao et al., 2021; Cheng and Houston, 2021). The discussion presented in this paragraph is based on cpDNA sequence information only. Tajima’s D values and the results of mismatch distribution analysis showed that the tested populations had not experienced obvious expansion events, and their population evolution was characterized by a model of recalcitrance to expansion (Figure 11; Supplementary Table 5). The results of STRUCTURE analysis showed that under the best *K* value of 2, more than 86.8% of the genetic components of all individuals of *C. drupifera* in the BQY, WSG, TJG, LJTL, HTDL, JX, ZX, LS, XYL, MNL, MP, and FH populations belonged to Cluster 2; similarly, more than 99% of the genetic components of individuals in the *C. meiocarpa* populations (HCX and CKX) came from Cluster 1, and 47.3 and 23.1% of the genetic components of the *C. oleifera* HZP and YML populations, respectively, came from Cluster 2. When *K* = 3 and *K* = 4, the populations of *C. oleifera* still showed a heterozygous composition of their genetic material, while the *C. meiocarpa* populations and the populations of Hainan Island presented a more homozygous composition (Figure 7). Thus, the *C. drupifera* populations from Hainan Island and the *C. meiocarpa* populations had a relatively uniform genetic background. The phylogenetic tree and PCA diagrams showed that the populations of *C. oleifera* were located between those of *C. drupifera* and *C. meiocarpa* (Figures 3, 5). In summary, we suggest that the *C. drupifera* population is a relatively primitive group in the context of the evolutionary relationships of the tested species, and *C. oleifera* may have evolved from the hybridization of *C. drupifera* and *C. meiocarpa*. Our field investigation showed that *C. meiocarpa* has small flowers, leaves and fruit, whereas most *C. drupifera* plants present large flowers, leaves and fruit, and *C. oleifera* presents an intermediate morphology. *C. oleifera* and *C. osmantha* are mostly hexaploid, *C. meiocarpa* is mostly tetraploid, and *C. drupifera* is mostly heptaploid or octoploid. These results also support our view regarding the evolution of *C. oleifera*. Further analysis of haplotype phylogenetic relationships also showed that the haplotypes of *C. oleifera*, *C. osmantha* and *C. meiocarpa* all evolved from the haplotypes of *C. drupifera*. H2 and H49 of *C. drupifer*a held the central positions among all the haplotypes in the TCS network (Figure 10).

Regardless of whether the analysis was based on ISSR and SRAP data or cpDNA data, when *K* = 2, 3, 4, or 5, most of the genetic components of the seven populations from Hainan Island belonged to an independent cluster (Figures 6, 7). This result implies that the population resources on Hainan Island represent a relatively homozygous *C. drupifera* group. The populations on Hainan Island were located at the edges of both the phylogenetic tree and the PCA map. We believe that the germplasm resources of *C. drupifera* originated from Hainan Island, China. After their introduction to mainland China, there was a high possibility of hybridization with other closely related species, forming a complex genetic group in mainland China. Due to the small number of populations and loci tested in this study, the relevant results need further study and verification.

The exchange of genetic material in plant species is an important driver of plant evolution (Stebbins, 1959; Soltis et al., 2014; Hübner et al., 2019; Steensels et al., 2021), whereas geographic isolation prevents gene exchange among populations (Gao et al., 2015; Di Santo and Hamilton, 2021). Based on the above analysis, we believe that interspecific hybridization caused by outbreeding and the high compatibility of tea-oil *Camellia* germplasm resources, the obstruction of gene communication caused by geographic distance, and artificial selection are important factors in the evolution of the species in Sect. *Oleifera*.

## Conclusion

According phylogenetic tree analysis, PCA and STRUCTURE analysis results, we believe that *C. oleifera.* var. *monosperma* is an independent species, with the suggested name *C. meiocarpa*. The analysis supported *C. gouchowensis* and *C. vietnamensis* were the same species with the unifying name *C. drupifera.* We further suggest that the tea-oil germplasm resources on Hainan Island represent an ecological type or a variant of *C. drupifera*, and *C. osmantha* is a new species in *Sect. Oleifera of the Camellia* genus showing the closest relationship to *C. drupifera*. Preliminary analysis showed that the tested populations did not experience obvious expansion events and indicated that *C. oleifera* may have evolved from the hybridization of *C. drupifera* and *C. meiocarpa*. *C. drupifera* was observed to be a relatively primitive group according to the evolutionary relationships of the tested species. The genetic diversity of the tested populations was low and the genetic differentiation among these populations was significant. The geographical distance factor played an important role in the genetic differentiation and gene exchange of the tested populations.

## Data availability statement

The original contributions presented in the study are included in the article/Supplementary material, further inquiries can be directed to the corresponding author.

## Author contributions

DZ designed and supervised the project. HQ, HY, DJ, XC, and DL prepared the samples and performed the experiments. HQ, XS, WY, JC, JY, CW, and TX analyzed the data. HQ wrote the manuscript. DZ and HQ revised the manuscript. All authors contributed to the article and approved the submitted version.

## Funding

The authors received specific funding for this work from the National Natural Science Foundation of China (31860082), the Project of Sanya Yazhou Bay Science and Technology City (SCKJ-JYRC-202258), the Science and Technology Innovation Project in the Hainan Academy of Agricultural Sciences (HAAS2022KJCX01), and the Project for Technology Development of Hainan Provincial Scientific Research Institutions (SQ2021JSKF0004 and SQKY2022-004).

## Conflict of interest

The authors declare that the research was conducted in the absence of any commercial or financial relationships that could be construed as a potential conflict of interest.

## Publisher’s note

All claims expressed in this article are solely those of the authors and do not necessarily represent those of their affiliated organizations, or those of the publisher, the editors and the reviewers. Any product that may be evaluated in this article, or claim that may be made by its manufacturer, is not guaranteed or endorsed by the publisher.

## Footnotes

^1^ http://www.fluxus-Engineering.com/
