# Supplementary material for: Genetic relationships and low diversity among the tea-oil Camellia species in Sect. Oleifera, a bulk woody oil crop in China
Source: Front Plant Sci. 2020 Sep 30;13:996731. doi: 10.3389/fpls.2022.996731 (PMC9563498; doi:10.3389/fpls.2022.996731)
Supplement: Supplementary file 1 [file Data_Sheet_1.docx]

***Supplementary Material***

***
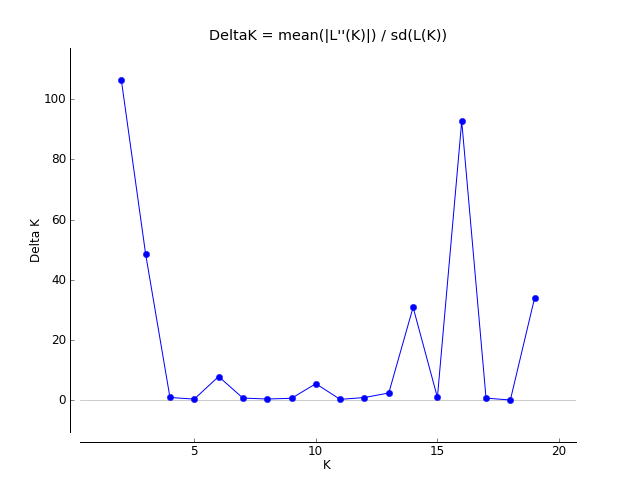
***

**Supplementary Figure 1. STRUCTURE HARVESTER analysis based on ISSR and SRAP data.**

***
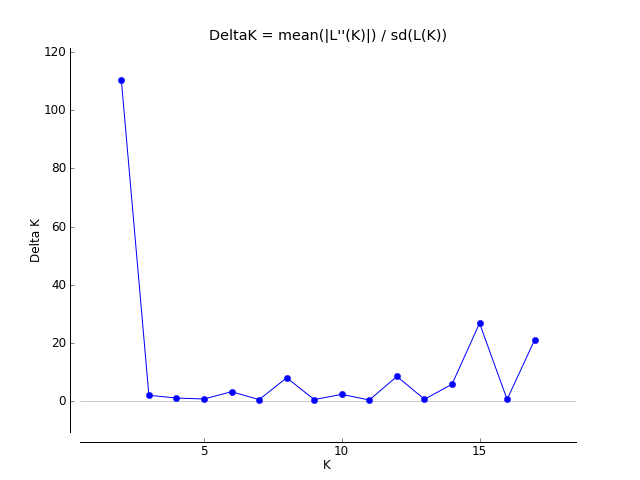
***

**Supplementary Figure 2. STRUCTURE HARVESTER analysis based on cpDNA data.**
